# Efficacy of 3-day low dose quinine plus clindamycin versus artemether-lumefantrine for the treatment of uncomplicated *Plasmodium falciparum* malaria in Kenyan children (CLINDAQUINE): an open-label randomized trial

**DOI:** 10.1186/s12936-022-04050-8

**Published:** 2022-02-02

**Authors:** Charles O. Obonyo, Elizabeth A. Juma, Vincent O. Were, Bernhards R. Ogutu

**Affiliations:** 1 Centre for Global Health Research, Kenya Medical Research Institute, P.O. Box 1578, Kisumu, Kenya; 2 Centre for Clinical Research, Kenya Medical Research Institute, P.O. Box 20778, Nairobi, Kenya

**Keywords:** Malaria, Clindamycin, Quinine, Artemether-lumefantrine, Children, Kenya

## Abstract

**Background:**

The World Health Organization recommends quinine plus clindamycin as first-line treatment of malaria in the first trimester of pregnancy and as a second-line treatment for uncomplicated falciparum malaria when artemisinin-based drug combinations are not available. The efficacy of quinine plus clindamycin was compared with that of artemether-lumefantrine in the treatment of uncomplicated *Plasmodium falciparum* malaria in children below 5 years of age.

**Methods:**

An open-label, phase 3, randomized trial was conducted in western Kenya. Children aged 6–59 months with uncomplicated falciparum malaria were randomly assigned (1:1) via a computer-generated randomization list to receive 3 days of twice a day treatment with either oral quinine (20 mg/kg/day) plus clindamycin (20 mg/kg/day) or artemether-lumefantrine (artemether 20 mg, lumefantrine 120 mg) as one (for those weighing 5–14 kg) or two (for those weighing 15–24 kg) tablets per dose. The primary outcome was a PCR-corrected rate of adequate clinical and parasitological response (ACPR) on day 28 in the per-protocol population.

**Results:**

Of the 384 children enrolled, 182/192 (94.8%) receiving quinine plus clindamycin and 171/192 (89.1%) receiving artemether-lumefantrine completed the study. The PCR-corrected ACPR rate was 44.0% (80 children) in the quinine plus clindamycin group and 97.1% (166 children) in the artemether-lumefantrine group (treatment difference − 53.1%, 95% CI − 43.5% to − 62.7%). At 72 h after starting treatment, 50.3% (94 children) in the quinine plus clindamycin group were still parasitaemic compared with 0.5% (1 child) in the artemether-lumefantrine group. Three cases of severe malaria were recorded as serious adverse events in the quinine plus clindamycin group.

**Conclusions:**

The study found no evidence to support the use of a 3-day low dose course of quinine plus clindamycin in the treatment of uncomplicated falciparum malaria in children under 5 years of age in Kenya, where artemether-lumefantrine is still effective.

*Trial Registration*: This trial is registered with the Pan-African Clinical Trials Registry, PACTR20129000419241.

## Background

Malaria is a major public health problem in sub-Saharan Africa. In 2018, malaria caused 405,000 deaths, globally, out of whom 67% were children below 5 years of age and 94% were residents of sub-Saharan Africa [1]. Universal implementation of artemisinin-based combination therapy (ACT) for malaria treatment and insecticide-treated bed nets for vector control comprises the main strategies for reducing malaria-related morbidity and mortality [2]. The World Health Organization (WHO) currently recommends five artemisinin-based combinations for the first-line treatment of uncomplicated *Plasmodium falciparum* infection, the most virulent and predominant malaria parasite in sub-Saharan Africa [2]. ACT is known to rapidly clear parasitaemia, delay the development of drug resistance, and reduce gametocyte carriage [3]. However, a high proportion of malaria cases in sub-Saharan Africa do not receive ACT due to factors associated with stock-out of drugs or poor access to healthcare providers [4]. In some settings, widespread deployment of ACT for malaria treatment has already resulted in significant reductions in malaria-related morbidity, mortality and admissions [5–8]. Artemisinin resistance (defined as delayed parasite clearance) has been reported in South-East Asia and most recently in sub-Saharan Africa [9–11]. Safe and effective alternatives to ACT are necessary.

In 2010, the WHO recommended second-line anti-malarial drug combinations with either an alternative ACT or a combination of quinine or artesunate with an antibiotic with anti-malarial activity (clindamycin, tetracycline, or doxycycline) [12]. However, data on the comparative efficacy between first-line and second-line anti-malarial combinations are scarce, as most research into anti-malarial drug efficacy has focused on comparing the efficacy of first-line treatments with an alternative artemisinin-based combination. The 2010 version of the WHO guidelines for the treatment of malaria recommended seven days of quinine plus clindamycin as a first-line treatment for malaria in the first trimester of pregnancy and as a second-line anti-malarial drug when ACT is not available [12]. The same guidelines recommended quinine for the treatment of severe falciparum malaria, uncomplicated malaria in pregnant women and drug-resistant malaria. Clindamycin is a lincosamide antibiotic with anti-malarial activities, used for the treatment of anaerobic and gram-positive bacterial infections, babesiosis, toxoplasmosis, and *Pneumocystis jirovecii* pneumonia [13]. Clindamycin is effective against *P. falciparum*, but it is a slow-acting drug with a mean parasite clearance time of four to six days and a mean fever clearance time of three to five days [14, 15]. In combination, the relatively fast action of quinine overcomes the drawback arising from the slow-action of clindamycin.

One systematic review of seven randomized trials found inconclusive evidence on the efficacy of quinine plus clindamycin compared with other anti-malarials (alone or in combination) in the treatment of uncomplicated falciparum malaria [16]. Another systematic review of 14 randomized trials found no difference in efficacy between quinine plus antibiotics compared with artemisinin-based and non-artemisinin-based combinations in the treatment of uncomplicated falciparum malaria [17]. There is no published study comparing the efficacy of quinine plus clindamycin with the recommended artemisinin-based combinations in the primary treatment of uncomplicated falciparum malaria.

In Kenya, the first-line anti-malarial for treatment of children and adults with uncomplicated falciparum is artemether-lumefantrine, which is generally well-tolerated and considered a highly effective fixed-dose anti-malarial drug combination. The efficacy and safety of quinine plus clindamycin was compared to that of artemether-lumefantrine in the treatment of uncomplicated falciparum malaria in children younger than 5 years of age in western Kenya.

## Methods

### Study design

This was an open-label, phase 3, randomized efficacy study to compare the rates of adequate clinical and parasitological response (ACPR) and safety between quinine plus clindamycin and artemether-lumefantrine in the treatment of uncomplicated falciparum malaria in Kenyan children aged below 5 years. This study was conducted at the outpatient clinics of Ahero sub-County Referral hospital (Kisumu County) and Homabay County Referral hospital (Homabay County), in western Kenya. The trial was conducted per the Declaration of Helsinki and Good Clinical Practice guidelines.

### Participants

Children were eligible for inclusion if they were aged six to 59 months, had an axillary temperature of 37.5^0^C or more or a history of fever in the past 24 h, microscopically-confirmed *P. falciparum* mono-infection and asexual parasite density of 2000 to 200,000 parasites/µL, ability to take oral medication, bodyweight below 50 kg and written informed consent by the accompanying parent/guardian. The study excluded children who had mixed plasmodial infection, a clear history of adequate anti-malarial treatment in the last 72 h, a history of allergy to artemisinin, clindamycin or quinine, evidence of severe malaria (according to standard definitions [2]), severe malnutrition (mid-upper arm circumference [MUAC] < 11.5 cm), or other concomitant febrile illness.

### Randomization and masking

Children were randomly assigned to receive either quinine plus clindamycin or artemether-lumefantrine, in a ratio of 1:1. Treatment allocation was made in blocks of eight according to a computer-generated randomization list by a statistician not associated with patient management. Sequentially numbered, sealed envelopes containing the treatment assignment were prepared according to the randomization list. Soon after inclusion, the study nurse allocated treatment by sequentially opening the envelope corresponding to the treatment number. The study was open-label, therefore, investigators and participants (or their parents or guardians) were aware of treatment allocation but laboratory technicians reading blood films were not aware of the study arm on which participants were allocated.

### Procedures

Children with suspected malaria during an outpatient visit were offered a screening blood smear test for malaria parasitaemia. Children who tested positive for malaria and met other study inclusion criteria were enrolled. At enrolment, a standardized medical history was taken and the children were clinically examined. Soon after randomization, children received the first directly observed dose of the study treatment. Children were admitted to the paediatric ward for three days to receive observed study treatment and for close monitoring.

Children assigned to the quinine plus clindamycin arm received 10 mg/kg of clindamycin (Cleocin paediatric® flavoured granules for oral suspension, Pfizer) administered twice daily (12 hourly) for three days as an oral suspension containing 75 mg/5 mL of clindamycin for a total daily dosage of 20 mg/kg of clindamycin. They also received 10 mg/kg of quinine (Universal Corporation Ltd), rounded to the nearest half tablet, administered twice daily (12 hourly) for three days as oral tablets containing 300 mg of quinine for a total daily dosage of 20 mg/kg of quinine. The quality of the clindamycin was certified by the US Federal Drug Administration, while the quality of quinine was certified by the Kenyan National Quality Control Laboratory, Nairobi. Children in the artemether-lumefantrine arm received the WHO-recommended weight-specific artemether-lumefantrine blister packs (Coartem; Novartis Pharma, Basel, Switzerland); The first artemether-lumefantrine dose was given at time 0 followed by a second dose 8 h later; on days 2 and 3, the child was treated twice per day. The dose of artemether-lumefantrine treatment was based on the child’s weight: 5–15 kg, 20 mg artemether + 120 mg lumefantrine; 15 to < 25 kg, 40 mg artemether + 240 mg lumefantrine. Administration of all the study drugs was directly observed by the study nurses. All the study drugs were dispersed in a small volume of water and dispensed by the study nurses. All children received milk 30 min before drug administration. Children were observed for 1 h after taking the drug to ensure retention; those who vomited within the first 30 min received a full repeat dose; those vomiting between 30–60 min received half the dose. Children with repeated vomiting were withdrawn from the study. Paracetamol syrup was administered to all children with temperatures ≥ 38.0 ℃.

Children were evaluated daily in the ward and 12-hourly blood slides were taken until two consecutive negative blood slides were obtained. Children were discharged home after they were clinically stable and had a negative slide. After discharge, the children were followed up for 28 days. Clinical reassessments were made on days 7, 14, 21, 28 and on any other day if the child was perceived to be unwell. During the follow-up visits, a standard medical history was taken, the axillary temperature recorded, physical examination performed, blood smears and filter paper for parasite genotyping taken. On days 0 and 28, a blood sample was taken for complete blood count and biochemistry. Post-treatment, children who developed signs of severe malaria were treated using parenteral artesunate or quinine; children with persistent parasitaemia or who developed recurrent parasitaemia without signs of severe malaria were treated using dihydroartemisinin-piperaquine (Duo-cotexcin; Beijing Holley-Cotec, Beijing, China) once daily for three days, according to the national malaria treatment guidelines. Children who could not continue with the study for any reason, including, inability to retain study medication due to repeated vomiting, progression to severe malaria, development of concomitant illness that could interfere with outcome classification, development of serious adverse events, ingestion of drugs with anti-malarial activities, withdrawal of consent or those who could not be traced, were withdrawn from the study. Adverse events and serious adverse events were assessed throughout the study and if found, were monitored until they resolved.

### Laboratory assessments

Capillary blood samples were obtained by finger prick at enrolment and follow up and were used to test for the presence of malaria parasites, determine haemoglobin (Hb) and for haematological and biochemical assessments. Thick and thin blood smears were prepared, stained with Giemsa and examined for malaria parasites. Parasite density was determined by counting the number of asexual parasites against 200 WBC in a thick smear. If *P. falciparum* gametocytes were detected, a gametocyte count was done per 500 leucocytes. Two microscopists independently read each smear, and parasite densities were computed by averaging the two counts. A third microscopist re-examined the smears if there were discordant readings with discordant results (difference in species or difference in parasite density > 50%).

The Hb level was measured using a portable HemoCue haemoglobinometer (HemoCue, Angelholm, Sweden). The haematology assessment was performed using Coulter Act Diff 2 Hematology Analyzer (Beckman Coulter, Brea, CA, USA) while the biochemical tests (alanine aminotransferase and creatinine) were done using a Reflotron Plus Chemistry Analyzer (Roche Diagnostics, Basel, Switzerland).

A dry filter paper blood spot was collected on day 0 and during follow up and used for parasite genotyping by polymerase chain reaction (PCR) analysis. To differentiate infections classified as recrudescence (same parasite strain) from a newly acquired infection (different parasite strain), a genotypic analysis based on merozoite surface protein-1 (*msp1)*, merozoite surface protein-2 (*msp2)* and glutamate-rich protein (*glurp)* was performed on paired filter paper blood samples (day 0 and day of recurrent parasitaemia) [18].

### Outcome classification

The primary efficacy endpoint was PCR-corrected adequate clinical and parasitological response (ACPR) on day 28 in the per-protocol population. ACPR is defined by WHO as the absence of parasitaemia on day 28, irrespective of axillary temperature, in a participant who has not previously met the criteria for early treatment failure, late clinical failure or late parasitological failure [2]. Secondary efficacy endpoints were assessed in the per-protocol population, and included the proportion of children with early treatment failure (with a modified definition to include presence of parasitaemia with or without signs of severe malaria), late parasitological failure and late clinical failure; the proportion of children with recrudescence or re-infection; the proportion of children with parasitaemia on day 2 and 3; the rate of gametocyte carriage; change in Hb from day 0 and the proportion of children with anaemia (Hb < 11 g/dL).

The safety endpoints included adverse events in children who had received at least one dose of the study medication. Only those events which occurred after the start of treatment or which worsened after starting treatment were considered. An adverse event was defined as any undesirable medical occurrence following administration of study treatment, irrespective of its causal relationship to the study medications. Adverse events were considered as serious if they were fatal, life-threatening, resulted in prolonged hospitalization, caused persistent/significant disability, or required specific medical or surgical intervention to prevent permanent impairment.

### Statistical analysis

With 80% power and a two-sided type I error of 0.05, we calculated that 167 children would be needed in each treatment group to detect a significant difference in ACPR rate, assuming a PCR-corrected ACPR rate of 97.4% with artemether-lumefantrine [19] and 90% with quinine plus clindamycin by day 28 after treatment [13]. An additional 25 children per treatment group were included to allow for loss to follow up and non-compliance. The total sample size was 384 (i.e., 192 per treatment group).

Data collected were recorded on paper-based case-record forms, entered into computers using Epi info (US Centers for Disease Control, Atlanta) and analyzed with SPSS for Windows (version 16.0) and Stata (version 14.0). We summarized the baseline characteristics using descriptive statistics. The efficacy was analysed using two methods: per-protocol analysis, where children who were withdrawn from the study or who were lost to follow-up were excluded from the analysis, and an intention to treat analysis, where all enrolled children are included in the analysis until the last day before drop-out.

Proportions were compared between treatment groups using the chi-squared test. For all comparisons, artemether-lumefantrine served as the reference group. Results are presented as risk differences, together with their 95% confidence intervals (CI). Normally distributed continuous variables were compared using the Student’s *t*-test. A two-tailed p-value less than 0.05 was considered statistically significant. For analysis of drug safety, the percentage of children who had each adverse event were compared between treatment groups.

## Results

### Participants

**Fig. 1:**
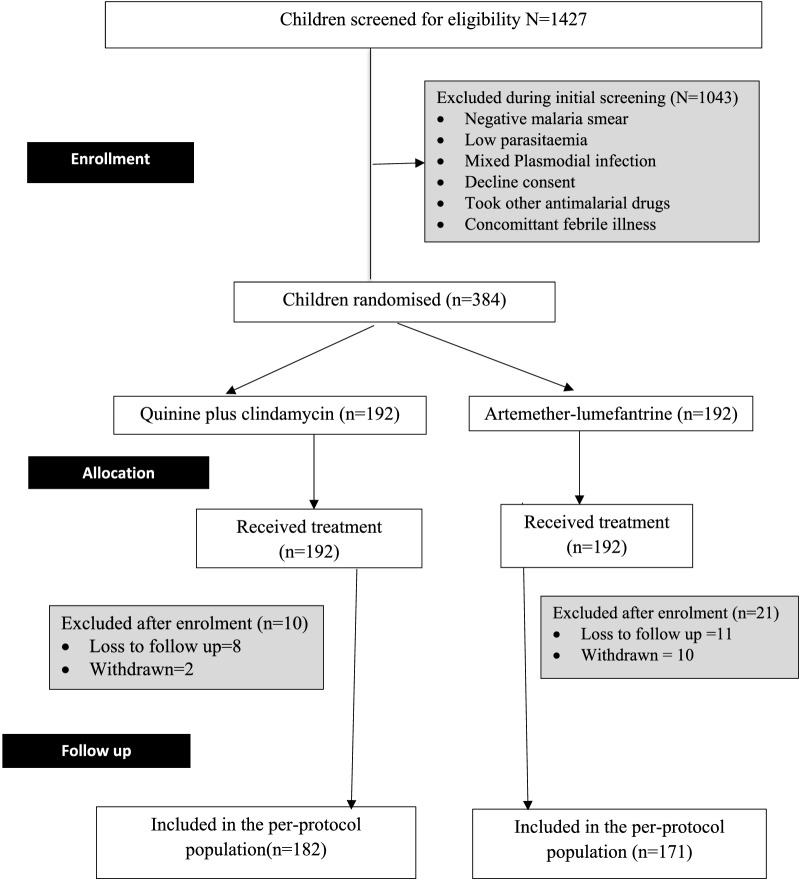
CONSORT trial chart

Between March 2014 and November 2014, a total of 1427 children were screened for eligibility; of these, 1043 were excluded for various reasons, including negative malaria smear, low parasite density, mixed plasmodial infections, recent ingestion of anti-malarial drugs, concomitant illnesses or lack of consent. A total of 384 children were enrolled and randomized equally to receive quinine plus clindamycin (n = 192) or artemether-lumefantrine (n = 192). At baseline, the children in both treatment groups were comparable on all variables that were measured, except for the proportion of anaemia which was significantly higher in the artemether-lumefantrine group (see Table 1). After enrollment, 4.2% (8/192) of children in the quinine plus clindamycin arm and 5.7% (11/192) in the artemether-lumefantrine arm were lost to follow up. Similarly, 1.0% (2 children) in the quinine plus clindamycin arm and 5.2% (10 children) in the artemether-lumefantrine arm were withdrawn from the study for various reasons. Thus, the primary outcomes were available for 94.8% (182 children) in the quinine plus clindamycin and 89.1% (171 children) in the artemether-lumefantrine arms, respectively (Fig. 1).

**Table 1:** Baseline characteristics of the study participants

Variable	Quinine plus clindamycin	Artemether-lumefantrine
Number	192	192
Study Centre		
Ahero sub-District Hospital	135	141
Homabay District Hospital	57	51
Mean age, months (SD)	31.7 (14.7)	33.2 (14.4)
Male sex (%)	98 (51.0%)	101 (52.6%)
Mean axillary Temperature (^o^C) (SD)	37.6 (1.0)	37.4 (1.0)
Patients with fever, ≥ 38.0 oC (%)	69 (35.9%)	55 (28.6%)
Median bodyweight (Kg) (IQR)	12.5 (6.0 to 20.0)	13.5 (6.5 to 24.0)
Mean haemoglobin (g/dL) (SD)	9.84(1.7)	9.84 (1.67)
Patients with anaemia (%)	134 (69.8%)	145 (75.5%)
Patients carrying gametocytes (%)	8 (4.2%)	6 (3.1%)
Geometric mean for asexual parasitaemia per µL (95%CI)	54,173 (45,794 to 64,084)	56,951 (48,813 to 66,447)

### Efficacy

The proportion of children with an adequate clinical and parasitological response (ACPR) was significantly lower in the quinine plus clindamycin group compared with the artemether-lumefantrine group, before and after adjusting the findings by genotyping. For the per-protocol population, the PCR-corrected ACPR was achieved in 44% (80/182) of the children (95% CI 36.8% to 51.2%) in the quinine plus clindamycin group and 97.1% (166/171) of the children (95% CI 94.6% to 99.6%) in the artemether-lumefantrine group (treatment difference – 53.1%, 95% CI -43.5% to – 62.7%). The PCR-corrected and PCR-uncorrected results were similar in the intention-to-treat population (see Table 2).

**Table 2:** Primary efficacy endpoints of quinine plus clindamycin and artemether-lumefantrine

	**Probability of cure**			
**Outcome**	Quinine plus clindamycin	Artemether-lumefantrine	Risk difference (95% CI)	P-value
Per protocol analysis				
PCR-uncorrected ACPR	58/182 (31.9%) [25.1% to 38.7%]	134/171 (78.4%) [72.2% to 84.6%]	− 46.5 [-36.1 to -56.9]	< 0.0001
PCR-corrected ACPR	80/182 (44.0%) [36.8% to 51.2%]	166/171 (97.1%) [94.6% to 99.6%]	− 53.1 [− 43.5 to − 62.7]	< 0.0001
Intention-to-treat analysis				
PCR-uncorrected ACPR	58/192 (30.2%) [23.7% to 36.7%]	134/192 (69.8%) [63.3% to 76.3%]	− 39.6 [− 29.6 to − 49.6]	< 0.0001
PCR-corrected ACPR	80/192 (41.7%) [34.7% to 48.7%]	166/192 (86.5%) [81.7% to 91.3%]	− 44.8 [− 35.2 to − 54.4]	< 0.0001

A significantly higher proportion of children in the quinine plus clindamycin group developed early treatment failure, 53.8% (98/182) compared with 0.6% (1/171) in the artemether-lumefantrine group (treatment difference 50.5%, 95% CI 43.3% to 57.6%) (see Table 3).

**Table 3:** Secondary efficacy outcomes of children with uncomplicated malaria after 28 days of follow up

	Quinine plus clindamycin N = 182	Artemether-lumefantrine N = 171
Early treatment failure	98/182 (53.8%)	1/171 (0.6%)
Late treatment failure	26 (14.3%)	36 (21.1%)
Due to recrudescence	4	4
Due to a new infection	22	32
Proportion of parasitaemic children		
Day 2	141/188 (75.0%)	21/189 (11.1%)
Day 3	94/187 (50.3%)	1/188 (0.5%)
Number of children with gametocytes who had no gametocytes on Day 0		
Day 7	10	1
Day 14	6	0
Day 21	3	0
Day 28	0	1
Mean Hb (g/dl) on day 28 (SD)	10.96 (1.47)	10.68 (1.30)
Mean increase in Hb on Day 28 (SE)[N]	1.12 (0.24) [N = 67]	0.84 (0.17) [N = 150]
Anaemia prevalence (Hb < 11 g/dl) on day 28 (%)	26/67 (38.8%)	68/150 (45.3%)

A total of 62 children developed recurrent parasitaemia between day 7 and day 28. Overall, 14.3% (26 children) of children on the quinine plus clindamycin arm compared with 21.1% (36 children) on the artemether-lumefantrine arm developed recurrent parasitaemia. 12.1% (22 children) of late treatment failure were categorized as re-infections on the quinine plus clindamycin arm compared with 18.7% (32 children) in the artemether-lumefantrine arm. Similar proportions (2%) of recrudescent infections were found between those on quinine plus clindamycin arm compared with those on the artemether-lumefantrine arm (see Table 3).

Parasite clearance was significantly slower in the quinine plus clindamycin group than in the artemether-lumefantrine group. By day 3, 50.3% (94/187) of the children in the quinine plus clindamycin group were parasitaemic compared with 0.5% (1/188) children in the artemether-lumefantrine group (difference 49.8%, 95%CI 42.6% to 57.0%). In both treatment groups, parasitaemia was cleared on day 7 (see Table 3).

In both treatment groups, the proportion of children with gametocytes decreased during follow up. However, this decrease was faster with artemether-lumefantrine compared to quinine plus clindamycin (see Table 3).

By day 28, the prevalence of anaemia had reduced by 31% (from 69.8% at enrollment to 38.8%) in the quinine plus clindamycin group and by 30.2% (from 75.5% at enrolment to 45.3%) in the artemether-lumefantrine group (p = 0.372) (Table 3). On day 28, the mean Hb concentration was 10.96 g/dl (SD 1.47) in the quinine plus clindamycin group and 10.68 g/dl (SD 1.29) in the artemether-lumefantrine group (z = 1.341, p = 0.179). The mean increase in Hb was significant within treatment groups but was not significantly different between the treatment groups (Table 4).

**Table 4:** Haematological and biochemical assessments

	Quinine plus clindamycin		Artemether-lumefantrine		P-value
	No. tested	Mean (SD)	No. tested	Mean (SD)	
White blood cell count (10^9^/L)					
Day 0	132	9.31 (4.2)	134	10.10 (7.8)	0.311
Day 28	51	7.92 (3.3)	118	7.71 (3.3)	0.701
P value	0.035		0.002		
Lymphocyte count (%)					
Day 0	131	30.06 (12.7)	133	34.5 (42.1)	0.252
Day 28	50	45.2 (14.7)	118	47.4 (13.5)	0.345
	< 0.0001		0.0016		
Red blood cell count (10^12^/L)					
Day 0	132	4.38 (4.3)	133	3.93 (1.00)	0.451
Day 28	51	4.56 (1.05)	118	4.39 (0.90)	0.275
	0.768		0.0002		
Haemoglobin concentration (g/dl)					
Day 0	131	9.64 (1.8)	133	9.8 (1.6)	0.425
Day 28	51	10.9 (1.5)	118	10.6 (1.2)	0.070
	< 0.0001		< 0.0001		
Alkaline phosphatase concentration (U/L)					
Day 0	164	21.1 (14.7)	164	20.5 (18.5)	0.738
Day 28	58	17.7 (8.7)	129	17.7 (7.7)	0.997
	0.098		0.108		
Creatinine concentration (µmol/L)					
Day 0	164	44.3 (16.5)	164	46.5 (31.9)	0.432
Day 28	58	49.2 (16.6)	129	46.1 (15.2)	0.219
	0.054		0.896		

### Safety

A total of 302 adverse event episodes were reported (Table 5). Overall, 74% (142/192) adverse events were observed in the quinine plus clindamycin group and 83% (160/192) in the artemether-lumefantrine group. The most common adverse events (> 5%) in the quinine plus clindamycin arm were anaemia, anorexia, cough, diarrhoea, runny nose, and weakness of the body, while on the artemether-lumefantrine, the most common were anaemia, anorexia, cough, diarrhoea, runny nose and skin rash.

**Table 5:** Adverse events

Adverse event	Quinine plus clindamycin N = 192 (%)	Artemether-lumefantrine N = 192 (%)
Anaemia	32 (17)	28 (14.6)
Abdominal pain	2 (1)	2 (1)
Anorexia	17 (8.9)	21 (10.9)
Cough	26 (13.5)	39 (20.3)
Diarrhoea	19 (9.9)	12 (6.3)
Itchy skin	4 (2.1)	2 (1)
Runny nose	12 (6.3)	36 (18.8)
Skin rash	6 (3.1)	10 (5.2)
Weakness of the body	14 (7.3)	7 (3.6)
Vomiting	7 (3.6)	3 (1.6)
Severe malaria	3 (2)	0
	142	160

Most of the adverse events were mild or moderate intensity. None of the adverse events was related to the study drugs. Three children treated using quinine plus clindamycin experienced a serious adverse event each. They all developed signs of severe malaria. All these children recovered completely after receiving treatment with intravenous artesunate with or without a blood transfusion for severe anaemia. There were no serious adverse events in the artemether-lumefantrine group. No child died in the study. Following the study treatment, measures of liver and kidney function did not change significantly between the treatment groups (Table 4).

## Discussion

Quinine plus clindamycin was significantly less effective than artemether-lumefantrine in the treatment of uncomplicated malaria in Kenyan children. Three days of treatment with quinine plus clindamycin was associated with a significantly low cure rate, a slower parasite clearance rate, a higher risk of early treatment failure and a greater predisposition to developing serious adverse events. Overall, this study does not support the treatment of young children with uncomplicated falciparum malaria using a short course of quinine plus clindamycin. Most of the recurrent infections in this study were due to re-infections, indicating a high malaria transmission in the study site.

Artemisinin-based combination therapy (ACT) is the recommended first-line treatment for patients diagnosed with uncomplicated falciparum malaria in all malaria-endemic regions. In 2010, the WHO recommended second-line treatment with a combination of quinine or artesunate with an antibiotic with anti-malarial activity [12]. However, PCR-corrected ACPR rates of 44% was found with quinine plus clindamycin and 97% with artemether-lumefantrine on day 28 after treatment of children with uncomplicated falciparum malaria in western Kenya. This is inconsistent with the findings of the QUINACT trial or the review by Song et al. [17, 20]. In the QUINACT trial, quinine plus clindamycin was administered for 5 to 7 days and had similar efficacy as artesunate plus amodiaquine and artemether-lumefantrine which were administered as rescue treatment for recurrent falciparum malaria in children [20]. A review comparing quinine-based with non-artemisinin-based and artemisinin-based anti-malarials found no significant difference in efficacy between quinine plus antibiotics compared with artemisinin-based combination treatments [17].

Some explanations for the low unexpected cure rates following treatment with quinine plus clindamycin can be posited. First, in this study, we gave quinine plus clindamycin treatment twice daily for three days based on a meta-analysis which found that a 3-day regimen of 12-hourly quinine plus clindamycin treatment was more effective than quinine alone, and on the assumption that a shorter treatment course would enhance compliance and minimize the incidence of adverse events [13]. The 2015, the WHO guidelines recommended that treatment with quinine plus clindamycin should be administered for seven days [2]. The same guidelines recommend that intravenous quinine should be given at a dose of 10 mg/kg three times a day (total daily dosage of 30 mg/kg) for 7 days. Lower doses of quinine (20 mg/kg/day, instead of 30 mg/kg/day) or shorter treatment courses have been associated with a lower treatment efficacy and lower risks of adverse events [21]. In the QUINACT study, quinine plus clindamycin was administered for 5 to 7 days and resulted in a higher efficacy compared to ACT which were given for 3 days [20]. It is unclear whether superior results would have been obtained by increasing the duration or frequency of treatment to 7 days. Secondly, the low cure rates observed in this study may have resulted from the low efficacy of quinine, suggested by reports of declining quinine efficacy in malaria-endemic areas [22–26]. Reduced sensitivity of malaria parasites to quinine therapy may result from easy accessibility and overuse. However, the efficacy of quinine in the treatment of uncomplicated falciparum malaria has not been evaluated in western Kenya. Lastly, the slow action of clindamycin may have contributed to the elevated risk of early treatment failure and reduced rate of ACPR. A total of 98 children treated with quinine plus clindamycin developed early treatment failure which was defined as parasitaemia on day 3, with or without signs of severe malaria. This may have resulted from the slow action of clindamycin with the subsequent delay in parasite and fever clearance [13].

Delayed clearance of malaria parasites by the third day after treatment is a strong predictor of treatment failure. By the third day after treatment, half of the children treated with quinine plus clindamycin in our study were still parasitaemic, consistent with the QUINACT trial that found a significantly slower rate of parasite clearance in children treated with quinine plus clindamycin compared to those who received ACT for recurrent falciparum malaria [20]. On day three, 52% (95/182) of children randomized to quinine plus clindamycin who were still parasitaemic were treated with dihydroartemisinin-piperaquine, as rescue treatment and they had complete parasite clearance by day 7. It is not clear whether the parasites would have cleared on day 7 without the rescue treatment. However, the risk of reinfection and recrudescence on the quinine plus clindamycin arm was possibly reduced by the post-treatment prophylaxis arising from the long half-life of piperaquine. In the revised edition of WHO guidelines (2015) for the treatment of malaria, based on expert opinion, quinine plus clindamycin was abandoned from the list of second-line treatments for uncomplicated falciparum malaria due to poor adherence associated with the 7-day treatment [2]. This study provides the evidence to support this decision.

Quinine plus clindamycin may be suitable for the treatment of uncomplicated falciparum malaria in children for whom tetracycline and doxycycline are contraindicated. However, the combination may be disadvantaged by the co-administration of the regimens, the complex dosing regime, the long duration of treatment, the cost and the limited availability of paediatric formulation of clindamycin [13]. The meta-analysis by Song et al. found that treatment with quinine plus antibiotics was associated with an increased risk of tinnitus and vomiting [17]. In our study, the combination of quinine with clindamycin was well tolerated and had a comparable safety profile to artemether-lumefantrine. These findings are similar to those of the QUINACT study [20, 27]. The relative safety and the low efficacy observed on the quinine plus clindamycin arm could be explained by the lower quinine dose that was administered over a short treatment period (three days). However, children who received quinine plus clindamycin were more likely to develop severe adverse events associated with worsening of the malaria infection.

Co-administration of quinine with clindamycin is the recommended first-line anti-malarial treatment in the first trimester of pregnancy [2]. However, in endemic areas, treatment of pregnant women with malaria in the first trimester has largely relied on quinine monotherapy due to the unavailability or cost of clindamycin [28]. Quinine therapy is known to be associated with low adherence due to tolerability (from bitter taste and adverse effects) and the need for multiple doses (three times a day) for 7 days [29, 30]. Interestingly, quinine plus clindamycin has not been evaluated in any clinical studies involving pregnant women in the first trimester. Despite insufficient safety data, the available efficacy data suggests that ACTs may be recommended in the treatment of confirmed malaria in the first trimester of pregnancy [31, 32].

In sub-Saharan Africa, artemether-lumefantrine is generally well tolerated and effective. On day 28, we found a PCR-corrected ACPR rate of 97% in children treated with artemether-lumefantrine. This confirms that in western Kenya, artemether-lumefantrine is still effective in treating children with uncomplicated falciparum malaria, but close monitoring of the efficacy of artemether-lumefantrine should continue. For treatment with artemisinin-based combinations, the WHO has recommended a change of treatment policy when the ACPR drops below 90%. In sub-Saharan Africa, ACPR rates below 90% have been reported for artemether-lumefantrine from Angola [33, 34], Gambia [35] and Malawi [36].

This study had the following limitations. The study was in western Kenya, meaning that the results may not apply to other malaria-endemic regions with different malaria transmission and drug resistance patterns. The study was open-label, suggesting that a remote susceptibility to bias may exist due to the awareness of participants and investigators of the treatment assignment. An active control (artemether-lumefantrine) arm was used, hence, a lower difference in ACPR was expected between the two treatment arms. This study was of short duration (28 days) and was not powered to detect statistically significant differences in adverse events. This study was limited to children with falciparum malaria. The results may, therefore, not apply to other *Plasmodium* species or adults. Finally, the implications of the unexpected low cure rate observed with quinine plus clindamycin are unclear, recalling that sample size was computed on the assumption of a 90% cure rate.

## Conclusion

This was the first randomized trial evaluating the efficacy of a low-dose, short course of quinine plus clindamycin compared with artemether-lumefantrine in the treatment of initial uncomplicated falciparum malaria infection in children below 5 years. The study found no evidence of a beneficial effect with a short treatment course of quinine plus clindamycin compared with artemether-lumefantrine for children with uncomplicated falciparum malaria. This study supports the decision by WHO to discourage the use of quinine plus clindamycin as a second-line treatment for uncomplicated falciparum malaria in children.

## Data Availability

The datasets used and/or analysed during the current study are available from the corresponding author on reasonable request.

## Abbreviations

ACPR: Adequate clinical and parasitological response
ACT: Artemisinin-based combination therapy
CI: Confidence interval
Glurp: Glutamte rich protein
g/dl: Grams per decilitre
Hb: Haemoglobin
IQR: Interquartile range
MSP: Merozoite surface protein
MUAC: Mid-upper arm circumference
N: Number
PCR: Polymerase chain reaction
SD: Standard deviation
SE: Standard error
WBC: White blood cells
WHO: World Health Organization
